# Building a framework for adolescent and young adult transition of care for patients with neurofibromatosis type 1 and neurofibromatosis type 2-related schwannomatosis syndromes

**DOI:** 10.3389/fonc.2026.1773687

**Published:** 2026-03-02

**Authors:** Daniel Schwartzbaum, Christine Dinh, Nicholas A. Borja, Aditi Dhir, Jennifer Coto, Diane Jung, Michelle Pei, Olivia Kalmanson, Bradley Gampel

**Affiliations:** 1 Department of Pediatrics, University of Miami Miller School of Medicine, Miami, FL, United States; 2 Sylvester Comprehensive Cancer Center, University of Miami Health System, Miami, FL, United States; 3 Department of Human Genetics, University of Miami Miller School of Medicine, Miami, FL, United States

**Keywords:** adolescent and young adult (AYA) cancer patients, cancer predisposition syndrome, health care transition, neurofibramatosis type 2 (NF2), neurofibramotosis type 1

## Abstract

Neurofibromatosis type 1 (NF1) and Neurofibromatosis Type 2-Related Schwannomatosis (NF2-SWN) are the two most common genetic disorders that present with Central nervous system(CNS) tumors. Patients with NF1 and NF2-SWN typically present in childhood and are initially managed by pediatric subspecialties. As CNS tumors are the leading cause of pediatric cancer related death, managing these tumors in NF1 and NF2-SWN patients requires coordinated multidisciplinary life-long care. Adolescent and young adult (AYA) transition of care is a major opportunity for improvement in NF2 and NF2-SWN outcomes. While few small studies have focused on the NF1 population, there is minimal literature covering NF2-SWN AYA transition of care. Healthcare transition (HCT) is extremely complex for all chronic patients; however, these complexities are magnified in patients with NF1 and NF2-SWN. Having an AYA HCT model can decrease gaps in care and improve survival. We review the literature on NF1 and NF2-SWN transition of care and describe the necessary framework to establish a system to improve patient outcomes.

## Introduction

Neurofibromatosis type 1 (NF1) and Neurofibromatosis Type 2-Related Schwannomatosis (*NF2*-SWN) are the two most common genetic disorders that present with Central nervous system (CNS) tumors. Patients with NF1 and *NF2*-SWN typically present in childhood and are initially managed by pediatric subspecialties. As CNS tumors are the leading cause of pediatric cancer related death, managing these tumors in NF1 and *NF2*-SWN patients requires coordinated multidisciplinary life-long care. Adolescent and young adult (AYA) transition of care is a major opportunity for improvement in NF2 and *NF2*-SWN outcomes. While few small studies have focused on the NF1 population, there is minimal literature covering *NF2*-SWN AYA transition of care. Healthcare transition (HCT) is extremely complex for all chronic patients; however, these complexities are magnified in patients with NF1 and *NF2*-SWN. Having an AYA HCT model can decrease gaps in care and improve survival (1, 2). We review the literature on NF1 and *NF2*-SWN transition of care and describe the necessary framework to establish a system to improve patient outcomes.

## A brief review of NF1 and *NF2*-SWN

NF1 remains one of the most prevalent RASopathies globally, with an estimated rate of 1/2,000-3,000 births (3). Diagnosis is established through characteristic clinical manifestations in combination with either a documented family history of NF1 or confirmatory molecular testing for germline or somatic pathogenic *NF1* variants (4). Among the pathognomonic features of NF1 are neurofibromas, peripheral nerve sheath tumors, and CNS tumors; which are typically benign however do carry the risk of malignant transformation. Management of neoplastic complications is tumor-specific and may involve surgical intervention, cytotoxic chemotherapy, or targeted agents such as MEK inhibitors acting along the RAS/MAPK signaling cascade (5).

*NF2*-SWN is an autosomal dominant disorder and tumor predisposition syndrome characterized by multiple benign tumors and dysplastic/hamartomatous lesions in the nervous system. *NF2*-SWN results from genetic loss of function in *NF2* gene causing loss of tumor suppression and dysregulation of multiple signaling pathways. Other forms of schwannomatosis will not be addressed individually in this review. The incidence of *NF2*-SWN is about 1 in 25,000-40,000 individuals. Patients typically present in adolescence and early adulthood with 18% diagnosed under 15 years old. Younger age at presentation can be associated with a more severe phenotype (6). Tumors and hamartomas associated with *NF2*-SWN affect all locations of the central and peripheral nervous system. Patients with *NF2*-SWN are at significantly increased risk of schwannomas, meningiomas, and ependymomas - over 95% of *NF2*-SWN patients will have vestibular schwannomas, and most *NF2*-SWN patients will become completely deaf. *NF2*-SWN patients also develop ophthalmic manifestations including juvenile cataracts (posterior subcapsular/cortical), epiretinal membranes (ERM), and retinal hamartomas, all of which threaten vision and thus create a need to prevent blindness. Other common manifestations of *NF2*-SWN are cutaneous conditions, with 70% of *NF2*-SWN individuals developing cutaneous schwannomas, subcutaneous nodules, and plaque-like lesions (7). While rare for the slow growing lesions to transform into high-grade tumors, the location and frequency of these tumors produce significant disease burden. Treatment options for *NF2*-SWN are limited. Surgical resection of *NF2*-associated tumors frequently leads to treatment-related neurological morbidity. Radiation therapy is used extremely cautiously due to the risk of malignant transformation, which is a particular concern in the *NF2*-SWN AYA population (8). There are currently no FDA-approved systemic therapies for NF2-associated tumors (9). Furthermore, auditory brainstem implant programs are limited in number, and not all patients benefit from cochlear implantation.

At a biological level, *NF1* and *NF2* function as tumor suppressors, which in the presence of a monoallelic, loss-of-function germline variant, confer predisposition to nervous system tumors, including schwannomas, meningiomas, and other peripheral and central nervous system neoplasms (10). Tumor burden and disease-related morbidity increase with age, and may lead to progressive neurologic impairment, chronic pain and functional decline. Importantly, each condition carries risk for more aggressive malignancy, such as malignant peripheral nerve sheath tumors in NF1 and high-grade meningiomas in *NF2*-SWN, reinforcing the need for lifelong surveillance for affected patients (1). Although both conditions are completely penetrant, they exhibit highly variable expressivity even within affected families (10). Further complicating this, both disorders have a high rate of *de novo* pathogenic variants, with somatic mosaicism accounting for a substantial proportion of sporadic cases and often leading to attenuated clinical manifestations (11). This phenotypic heterogeneity can delay diagnosis and complicate clinical recognition and appropriate surveillance. Moreover, as affected individuals enter adulthood and begin family planning, the autosomal dominant inheritance pattern of these conditions becomes increasingly relevant, as individuals with NF1 or *NF2*-SWN have a 50% risk of transmitting the condition to each offspring, underscoring the need for genetic counseling across multiple life stages (10).

## The need for AYA healthcare transition in NF1 and *NF2*-SWN

NF1 and *NF2*-SWN share fundamental clinical features with important implications for the healthcare transition including the complexity and chronicity of manifestations (12). Regarding NF1 specifically, the risk of developing specific tumors may vary throughout childhood and adolescence, warranting a robust surveillance protocol. Current recommendations include annual ophthalmic exams including fundoscopy and optical coherence tomography until age 8 years; a one-time whole-body magnetic resonance imaging (MRI) during adolescence to assess for high-risk neurofibroma, or prior to starting MEK inhibitor; and fludeoxyglucose-18 positron emission tomography (FDG-PET) when malignant peripheral nerve sheath tumor is suspected. Given that cumulative tumor risk peaks in the AYA period, uninterrupted surveillance through a well-coordinated transition to adult care is essential.

For AYAs with NF1, the presence of physically deforming skin lesions often exposes patients to bullying and social stigmatization (13), resulting in significant mental distress, higher rates of anxiety and depression, and worse QOL (14, 15). In addition to social stressors, other predictors for QOL in AYAs with NF1 include degree of health literacy, severity of pain symptoms, ability to perform activities of daily living, and cognitive functioning (9, 16). NF1 plexiform neurofibromas frequently result in significant and chronic pain which interferes with daily functioning (17), and is associated with poorer quality of life (QOL), functional limitations, and increased emotional distress (18). Conversely, increased anxiety predicts greater pain-related interference in activities of daily living. Addressing psychological distress may improve mental health outcomes while also reducing pain burden and enhancing functional capacity.

Cognitive impairment is a pervasive challenge for AYA patients with NF1 (3). These difficulties, which persist into adulthood manifest as impairments in attention, executive functioning, working memory, and learning, contributing to academic underachievement, reduced independence, and increased psychosocial vulnerability (14, 19). Beyond academic challenges, studies indicate that individuals with NF1 also experience difficulties in social-cognitive processing, such as encoding tasks involving facial recognition and emotion identification (20). Difficulties with social skills may impair relationship building and increase social isolation, compounding existing psychosocial stressors such as appearance related bullying (21). Social isolation and bullying can exacerbate low self-esteem and levels of stress in patients with NF1 (22).

Comparatively, *NF2*-SWN often manifests as a myriad of neurological symptoms, from hearing loss (HL) to intractable pain caused by schwannomatosis (7). The broad distribution of tumor locations warrants coordinated care by multiple subspecialists ideally at a specialized *NF2*-SWN center (3). Subspecialities include but are not limited to genetics, neurology, neurosurgery, otolaryngology, neuro-oncology, hematology-oncology, ophthalmology, dermatology, audiology, neuropsychology, pain specialists, orthopedics, and neuro-radiology. As these tumors are predominantly slow growing, the providers’ goal is to preserve life, minimize toxicity from the disease, minimize toxicity from disease treatments, and improve QOL. Greater symptom severity has been associated with reduced QOL in adults with NF2-SWN (19). Studies in adults further suggest that psychological resilience may buffer the emotional and psychosocial distress associated with *NF2*-SWN, and that interventions aimed at enhancing resilience may improve QOL (20, 23). AYAs with *NF2*-SWN experience a gradual decline in functional independence with increased reliance on support for activities of daily living. This distress can be further exacerbated if patients also recognize the overall poor prognosis of their condition; historically the average age at death is 36 years old (24), however that average may me lower in the most severe phenotypes (11).

## Current barriers to AYA healthcare transition in NF1 and *NF2*-SWN

**Figure 1:**
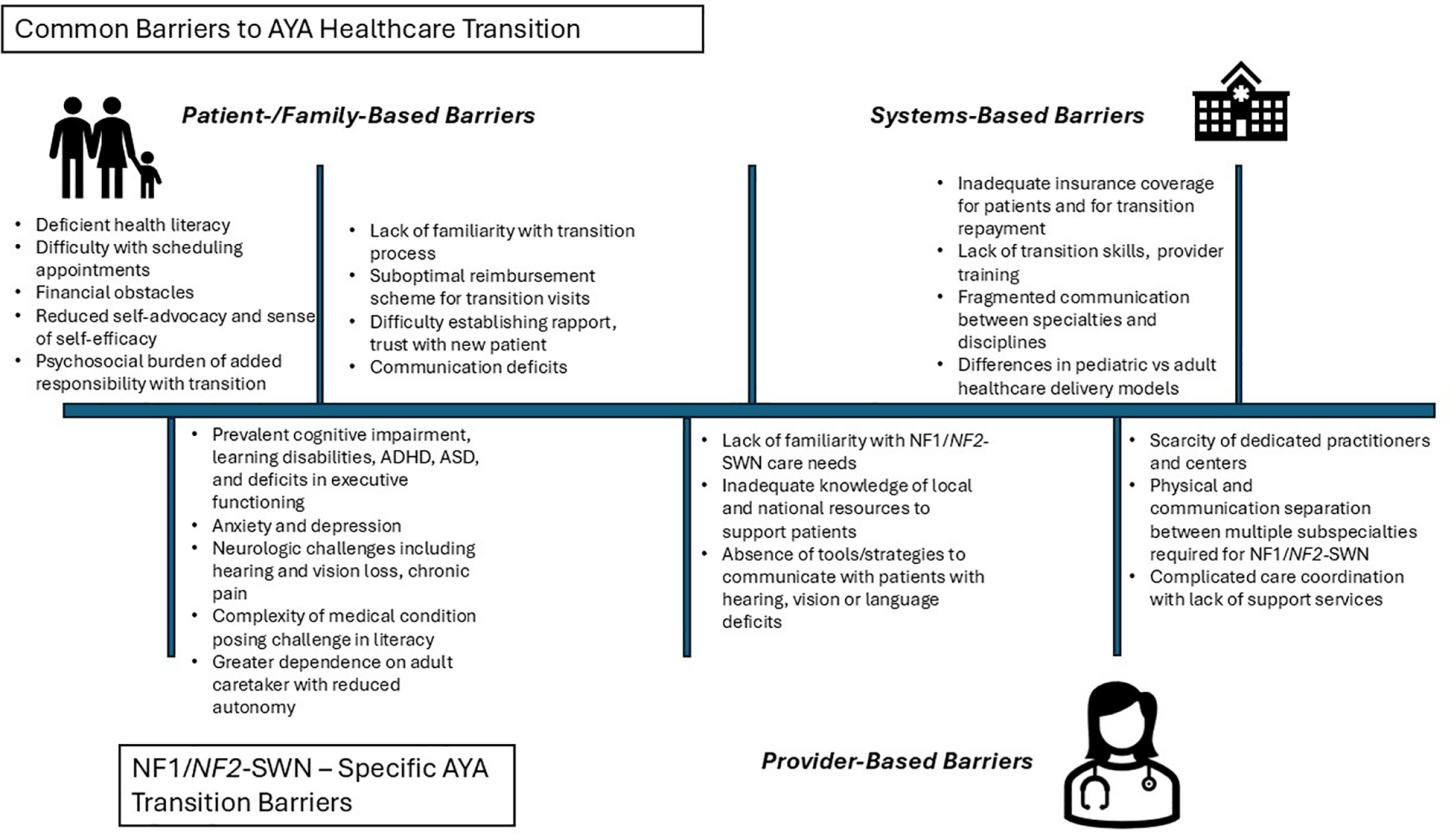
Detailing both common barriers to health care transition for all adolescent and young adult patients as well as barriers that are specific to patientswith Neurofibromatosis 1 and Neurofibromatosis Type 2-Related Schwannomatosis.

While the importance of AYA Healthcare transition for NF1 and *NF2*-SWN patients is evident, there remains significant obstacles (**Figure 1**). The medical and psychosocial complexities of these patients create challenges to successful healthcare transition and thus requires early and deliberate planning (1, 25). Patients with NF1 and *NF2*-SWN are often classified as children and youth with special healthcare needs (CYSHCN) (26) AYA patients with NF1 and NF2-SWN, report similar HCT challenges as other CYSHCN groups. Barriers include knowledge of medical conditions, access/affordability of new providers, provider and systems-based deficits in transition skills, staffing, resources, provider expertise, and shared patient-provider communication and trust.

Compared with other childhood-onset conditions, fewer pediatric medical centers are equipped to provide specialized care for NF1 patients, and even fewer adult medical centers (27). NF1 patients’ understanding of their condition is often limited due to both the complexity of their disease, and by a higher prevalence of neurocognitive impairments. These knowledge gaps have significant implications for autonomy, family planning, and reproductive decision-making. Individuals with NF1 also experience higher rates of psychological comorbidities, including anxiety and depression, related to chronic symptom burden and disease uncertainty, which can contribute to social isolation and in turn result in challenges with regular healthcare provider follow-up.

Similar to NF1, access to high-quality, coordinated care for AYAs with *NF2*-SWN is limited by disease-specific barriers including sensory impairments, neurological morbidity, and high healthcare utilization. These factors underscore the urgent need for a multidisciplinary, developmentally informed transition pathway to optimize clinical and psychosocial outcomes. *NF2*-SWN affects multiple neurologic and sensory systems, profoundly altering how individuals experience and interact with the world. Patients are at high risk for progressive HL often culminating in deafness, as well as facial paralysis, vestibular dysfunction, voice and swallowing difficulties, chronic pain, and limb weakness (28). As symptoms progress, many individuals with *NF2*-SWN struggle to achieve independence from their families, placing strain on critical support relationships (29). Ongoing tumor surveillance with MRI can be complicated by hearing implant-related artifacts or the need for surgical magnet removal (30). *NF2*-SWN individuals often rely on additional modes of communication, including sign language, lip reading, or text-based messaging (31), which can further impede employment and independent management of healthcare tasks. Within this context of functional decline, uncertainty about prognosis, and relational stress, individuals with *NF2*-SWN experience higher rates of depression and anxiety compared to the general population (32), with depression also associated with increased pain intensity (34, 35). In turn, depression and anxiety may exacerbate relational strain, particularly with caregivers on whom patients depend. These factors underscore the need for a multidisciplinary, developmentally informed transition pathway to optimize clinical and psychosocial outcomes.

While peers are gaining educational, vocational, and financial independence and forming long-term relationships, NF1 and *NF2*-SWN AYA patients must spend substantial time attending frequent medical visits to multiple specialists. This bears the risk of imposing financial barriers to accessing needed care and producing a vicious cycle of disrupted care. Additionally, many NF1 and *NF2*-SWN AYA patients experience insurance instability as they lose coverage after aging out of parental insurance plans, particularly when disease-related disability limits full-time employment. Employment and financial barriers also arise from the direct consequences of disease manifestations in both conditions. Cognitive impairments that are frequently comorbid in NF1 can produce challenges in education and vocational training needed for long-term gainful employment. Meanwhile, the sensory and neurological impairments seen in *NF2*-SWN create various barriers to workforce participation. HL and the variable outcomes of cochlear and auditory brainstem implants can limit employment opportunities; employers may be unable to accommodate alternative means of communication. Facial paralysis represents an additional obstacle to social and occupational functioning, as facial weakness is associated with lower perceived trustworthiness in the general population (33). Chronic pain, extremity weakness, imbalance, and dizziness further restrict functional capacity and job retention, especially in professions that require employees to be able to walk or perform more physically strenuous activity. Furthermore, vocal disorders can significantly impact daily activities, reduce work productivity, and increase absenteeism, particularly in teachers, sales personnel, and customer service workers in call centers (34).

An effective HCT for young adults with NF1 and *NF2*-SWN is often limited by knowledge gaps among adult providers (1, 35). While pediatric care is often delivered through specialized, multidisciplinary clinics with specific surveillance protocols, many providers in adult care settings have limited familiarity with the natural history and lifelong tumor risks of these rare conditions (36, 37). In particular, the incorrect expectation that disease manifestations will stabilize after childhood can result in the under recognition of morbidity, as well as failure to initiate the appropriate surveillance. For instance, providers must be aware that their adult female patients with NF1 should be guided to breast MRI screening beginning at age 30 as women with NF1 < 50 years old have a five-fold increased risk of disease (37, 38).

The transition to adulthood for these patients is also complicated by the significant fragmentation that exists across medical specialties, including but not limited to neurology, ophthalmology, oncology, otolaryngology, neurosurgery, and medical genetics, often without any single provider well positioned to coordinate care (1, 36). These obstacles are exacerbated by the lack of medical training programs providing formal education on management of inherited CNS tumor predisposition syndromes in adults, which contributes to poor adherence to consensus surveillance recommendations (35, 37). The frequency with which clinical guidelines are revised adds further difficulty to the delivery of evidence-based care (1, 3).

## Creating a framework for AYA NF1 and *NF2*-SWN healthcare transition

There is no agreed upon standard of care HCT for AYA patients with NF1 and *NF2*-SWN. All models have used NF1 as a template with no published models focusing on *NF2*-SWN. Recent studies with NF1 AYA HCT demonstrated that starting transition preparation as early as 13 years old and providing patients with portable medical summaries led to increased success in transition. Another NF1 study emphasized the importance of a structured transition system as a quarter of their patients were never transitioned to an adult center. Incorporating transition specialists, whether nurses or advanced practitioners, familiar with disease complexities improved HCT success (39, 40). Other programs offer patient resources with roadmaps detailing upcoming appointments and transition steps, using specialized psychotherapy and mobile phone software (41, 42). The literature clearly supports additional support for patients with NF1 and *NF2*-SWN to facilitate transition (43). However, long-term evaluation of prior NF specific HCT interventions and outcomes remains limited.

**Figure 2:**
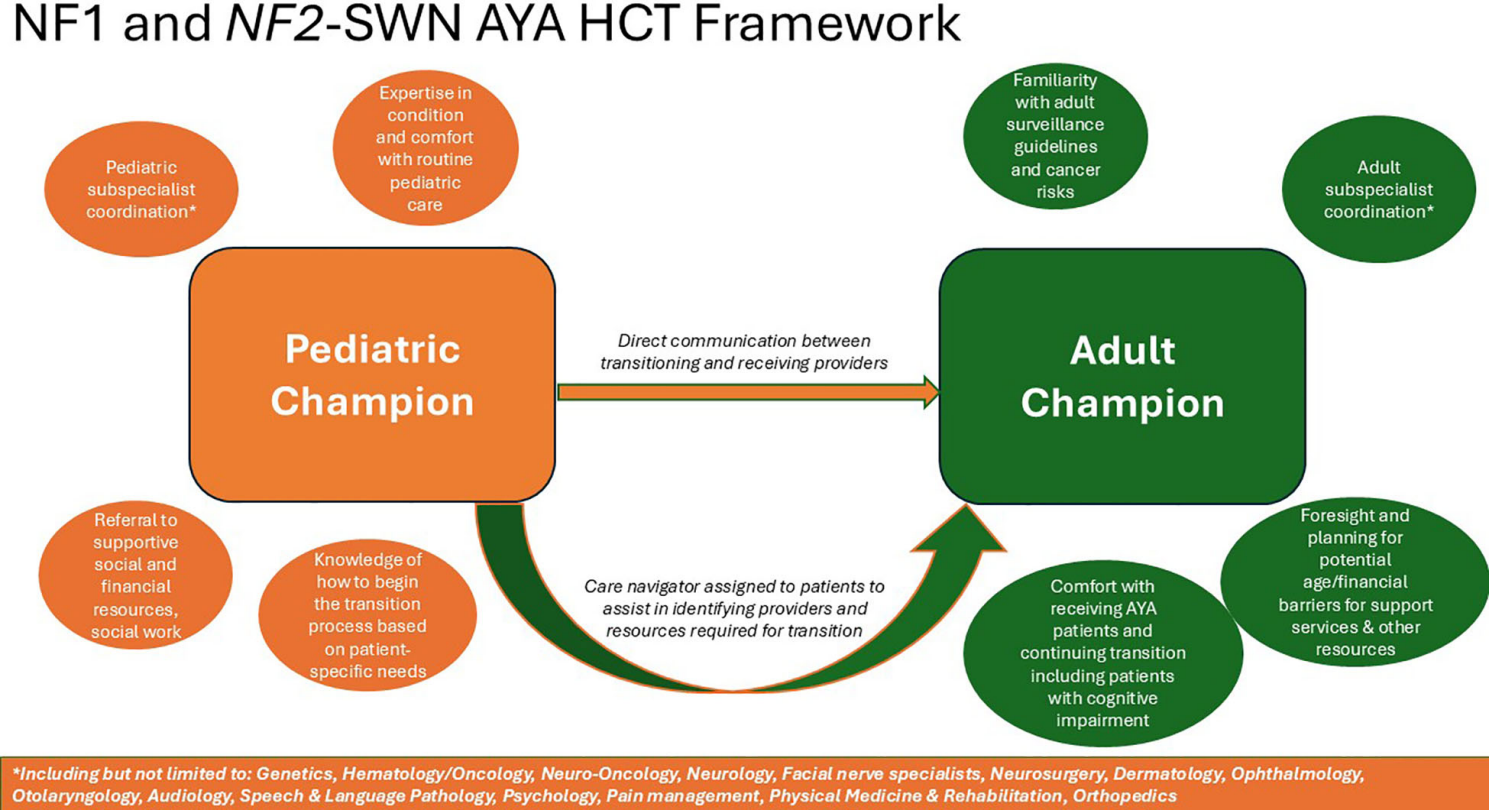
Model healthcare transition framework for patients with Neurofibromatosis 1 and Neurofibromatosis Type 2-Related Schwannomatosis.

Evaluating other chronic conditions that present in childhood, we find common themes in HCT frameworks (**Figure 2**). For pediatric CNS patients without cancer predisposition syndromes, the literature reinforces the need for a systematized manner of transition, with more emphasis on having an overall structure than the specific model (44–46). Programs incorporating multidisciplinary and longitudinal care performed better. The most frequent types used were “adult caregiver model” where patients transition from pediatric to adult care and “joint caregiver model” involving consultation with pediatric and adult specialists. The optimal model for NF1 patients in one study was the “specialized clinic model” with patients managed lifelong in a specialized clinic (47). Looking at all childhood cancers, we find similar results, that transition tools, patient disease specific education, adult provider expertise, and transition provider communication are required (48–50). For patients with type 1 diabetes, again the most important components were a structured program with care coordination support as opposed to a specific model (51). In sickle cell disease, perhaps the most similar to NF for HCT, one recent study demonstrated that educating adult caregivers, identifying champions, and sharing health record were keys to successful HCT programs (52).

## Discussion

NF1 and *NF2*-SWN are CNS tumor predisposition syndromes that require lifelong complex multidisciplinary care. While NF patients experience common AYA HCT hurdles, these are compounded by neurocognitive deficits, chronic pain, vision loss, HL, and disease understanding. Symptoms progress as patients age leading to functional decline in activities of daily living, social isolation, anxiety/depression, and reduction in QOL (6, 15). Clinical phenotypes are heterogeneous, requiring individualized approaches.

Substantial opportunities exist to improve AYA and adult outcomes across the NF1 and *NF2*-SWN disease trajectory. Recent studies highlight ways to improve patient access, reduce patients lost to follow-up, and the importance of educating adult providers (27, 53). Disruptions in care can delay identification of disease progression and access to rehabilitative and psychosocial support, leading to worse morbidity and mortality (54, 55). In *NF2*-SWN, earlier hearing rehabilitation, particularly advances in auditory brainstem implants, should be an area of focus for HCTs (7, 54, 55).

Analyzing prior NF1 studies, as well as other chronic conditions presenting in childhood, common patterns emerge to build an optimal HCT framework. Programs need coordinated multidisciplinary care to optimize timing and execution of transition across the care continuum, addressing both the complex medical needs and the psychosocial challenges. While the exact patient age to start is undetermined, the process must be introduced in early adolescence to prepare patients and caregivers. A “champion” to coordinate the transition, whether nurse, nurse practitioner, social worker, or physician should be identified within both the pediatric and adult sides. These champions must then work very closely with the subspecialists in all core disciplines. Ideally, the adult provider works within an NF center affiliated with an academic medical institution to provide more disease-specific resources. Just as important is distinguishing adult subspecialists experienced in NF1 and *NF2*-SWN who have the capability and willingness to accept these patients. Focused patient and caregiver education, as well as structured protocols, can improve communication between teams. Equally important for providers is a procedure for the exchange of all healthcare information. Each HCT also needs a dedicated care navigator to ensure subspecialist appointments are made in a timely manner. Each HCT must involve social workers to mitigate financial toxicity. The framework for NF1 and *NF2*-SWN HCT programs must be individualized based on resources, geographical constraints, and nearby specialists. Providers can take an individualized approach based on each patient’s distinct needs and circumstances to assess transition readiness, identify accessible resources, and accepting adult institutions. Buy-in is needed from all stakeholders including pediatric providers, adult providers, patients and their caregivers (**Figure 2**).

AYA HCT for NF1 and *NF2*-SWN patients has the potential to succeed within the appropriate framework. Pediatric and adult providers caring for these patients must be prepared to address the myriad of transition challenges via careful care coordination recognition of disease- and patient-specific needs, and knowledge of available resources and specialists. While improving AYA transition of care in NF1 and *NF2*-SWN is feasible, coordinated future prospective studies must be undertaken to establish a standard of care and improve morbidity and mortality.
